# Teaching in Uncertain Times: Expanding the Scope of Extraneous Cognitive Load in the Cognitive Load Theory

**DOI:** 10.3389/fpsyg.2022.665835

**Published:** 2022-06-24

**Authors:** Tracey A. H. Taylor, Suzan Kamel-ElSayed, James F. Grogan, Inaya Hajj Hussein, Sarah Lerchenfeldt, Changiz Mohiyeddini

**Affiliations:** Department of Foundational Medical Studies, Oakland University William Beaumont School of Medicine, Rochester, MI, United States

**Keywords:** COVID-19, pandemic, Cognitive Load Theory, intrinsic load, extraneous load, germane load, the impact of the situation

## Abstract

The COVID-19 pandemic caused an unprecedented and highly threatening, constrained, and confusing social and educational environment, we decided to expand the traditional focus of the extraneous load in Cognitive Load Theory (CLT) acknowledging the psychological environment in which learning occurs. We therefore adapted and implemented principles of the CLT to reduce extraneous load for our students by facilitating their educational activities. Given previous empirical support for the principles of CLT, it was expected that the adoption of these principles might enable our students to cultivate attitudes and skills across multiple domains such as online learning and presentation technologies, implementing and maintaining a “classroom atmosphere” in a virtual environment, participating in discussions among large online groups of students, facilitating group work, providing virtual office hours for students, and proactively planning for upcoming semesters.

## Introduction

The onset of the COVID-19 pandemic caused unprecedented levels of disruption upon all areas of life worldwide (Castelnuovo et al., 2020). Health professions educators struggled to determine the most efficient way to teach students during these times when conventional teaching methods became unexpectedly unavailable. Our goal is to reduce extraneous load for students by adapting and implementing principles of the Cognitive Load Theory (CLT; Sweller, 1988, 2010), especially during times of crisis, such as the uncertain times experienced during the pandemic of SARS-CoV-2.

Rapidly developing educational theories, instruction methods and presentation techniques are enhancing our ability to understand human learning behavior, performance and outcomes, and indeed, how we define learning and its constituents (Ten Cate et al., 2011; Yardley et al., 2012; Khalil and Elkhider, 2016; McInerney and Green-Thompson, 2017; Barbour and Schuessler, 2019; Kardong-Edgren et al., 2019). In this line of research, CLT asserts that humans possess a remarkably adaptive, yet limited, cognitive capacity to learn. Hence, according to CLT, a key strategy to foster learning is to efficiently regulate how this limited cognitive capacity is deployed.

Relying on Atkinson and Shiffrin’s (1968) model of human memory, CLT postulates three cognitive components of the human learning architecture: a sensory memory (SM), working memory (WM), and a long-term memory (LTM). SM is assumed to be confined to perception of visual and auditory information. However, it can retain information for only a few seconds (Mayer, 2010; Eriksson et al., 2015). Because only information that rises to awareness can enter the domain of the WM, most of the information entering SM vanishes and only a small portion of the sensory information enters the domain of WM (Simons and Chabris, 1999). CLT assumes that WM displays a limited capacity in dealing with complex, novel, and unorganized information elements obtained through SM. WM can only hold five to nine information elements (the famous “seven plus or minus two”) (Miller, 1956), which vanish after a few and up to about 20 s, unless they are revitalized by rehearsal (Fegen et al., 2015; Lucidi et al., 2016; De Schrijver and Barrouillet, 2017; Oberauer, 2019). Furthermore, CLT asserts that the capacity and the characteristic of the working memory is a function of the type of information elements and varies depending upon whether the information elements are novel or have been retrieved from LTM (Sweller, 2010; van Merriënboer and Sweller, 2010).

In contrast to WM, LTM has no known capacity limitations (Witt et al., 2019) and holds cognitive schemas that assist LTM by organizing information (Sporer, 2016). Cognitive schemas vary in their degree of complexity and reduce the amount of information units that are processed concurrently during the problem-solving process, for instance, by clustering new elements together or by integrating new elements in schemas already available in LTM (Anderson, 1980; van den Bosch and Daelemans, 2013; Wirzberger et al., 2018). Frequent reactivation and successful application of a schema leads to automatization and significantly reduces the burden of information processing (Gobet, 2000). According to CLT, the process of construction and automatization of cognitive schemas constitutes learning (van Merriënboer and Sweller, 2010). Therefore, efficient, and successful learning requires an ease in the process of creating and modifying cognitive schemas to optimize intrinsic and extraneous cognitive load for upcoming learning to levels that do not exceed the learner’s cognitive capacity and do not impede learning performance of an individual (Reif, 2010).

According to CLT, individuals must deal with three different, but interdependent types of the cognitive load in the process of learning: intrinsic, extraneous, and germane load (Sweller, 1988, 2010). Intrinsic cognitive load originates in the content of information elements and intrinsic nature of the learning tasks. It increases by the number, complexity, and novelty of information elements that must be processed simultaneously in working memory. Simultaneous processing of information depends on the extent of element interactivity. Element refers to a cue (e.g., a piece of information) that has been or needs to be learned (Sweller et al., 1988). Tasks with low Low-element interactivity (e.g., the vocabulary of a foreign language) allow elements to be learned serially rather than simultaneously whereas learning tasks with high-element interactivity requires that several elements must be manipulated in working memory simultaneously (unthreatening a sentence requires understanding all of the words in that sentence). Apart from the complexity and novelty of information element content, it is also decisive how this new information element is presented to the individual (extraneous load). Accordingly, extraneous load is created by instructional procedures. A central premise of CLT alleges that those intrinsic and extraneous cognitive loads are additive. Consequently, in the presence of high intrinsic cognitive load, it would be decisive to reduce the extraneous cognitive load by applying efficient and user-friendly instructional procedures (Sweller, 1994; van Merriënboer and Sweller, 2010; Naismith et al., 2015; Jordan et al., 2020). In contrast to intrinsic and extraneous cognitive load, germane load, refers to the cognitive load that is required to create cognitive schemas and depends on the amount of WM resources used to regulate and deal with intrinsic cognitive load.

## Expanding the Scope of Extraneous Cognitive Load

Despite abundant work on extraneous cognitive load (Klepsch et al., 2017; Sewell et al., 2019), one piece of research seems to be surprisingly missing in the literature as learning and instructional procedures do not occur in a situational vacuum. This assertion challenges the substantive definition of extraneous cognitive load as it assumes that situational characteristics may ease or deteriorate the process of development, modification, and automatization of cognitive schemas over and above the quality of the instructional process. Therefore, the concept of “extraneous cognitive load” must encompass the broad diversity and complexity of situational factors under which learning materials are presented. We sought to address this limitation in the present work.

The issue of efficient use of cognitive resources pertains to medical students. Medical education follows a highly challenging, fast paced, and dense curriculum (Stevens, 2018; Buja, 2019). Previous research has established that independent of myriad daily life stressors, college life under *normal* circumstances is an extremely stressful and critical period (Anderson et al., 2003; Hamilton, 2006). Medical students have been consistently reporting high levels of stress, anxiety, and depression (Iqbal et al., 2015; Ludwig et al., 2015; Brenneisen Mayer et al., 2016; Rotenstein et al., 2016; Fawzy and Hamed, 2017; Moutinho et al., 2017). However, it is crucial to emphasize that online education was well-established prior to the COVID-19 pandemic (Castro and Tumibay, 2021) and online instruction has been ubiquitous in medical education (Jiang et al., 2021). However, COVID-19 imposed an unprecedented pressure on medical students to adapt rapidly to a sudden pivot from traditional face-to-face instruction to online instruction without any lead-time to prepare for such a massive change. Therefore, it was crucial to identify and prevent potential sources of cognitive overload for our students to support the learning performance and wellbeing of our students. Hence, in times of a health pandemic such as the current COVID-19, it seems particularly plausible to assume that an abrupt shift to online teaching elevates anxiety and fear among students and exacerbates efficient use of their cognitive resources.

Given the urgency of the COVID-19 pandemic, and in the absence of any prior experience and evidence-based recommendations for how to support and enhance learning experiences of all students by shifting to online instruction, we decided to apply CLT to enhance students’ learning capabilities. CLT is a well-known theory that uses cognitive structures such as working memory and long-term memory to explain human learning. This decision was encouraged by empirical evidence in the past supporting the principles of CLT during non-pandemic times (van Merriënboer and Sweller, 2010; Sweller et al., 2011, 2019).

Therefore, we have adapted several theory-based strategies to ease the burden of learning for our students and to train them to become lifelong learners during this time of crisis. Many of these strategies focus on aiding students to acquire the skills needed for problem-solving and self-assessment and build on their existing knowledge.

The strategies that are presented in this paper are the outcome of the reflections of our team in dealing with COVID-19 pandemic challenges to prepare for online-only instruction. In addition, we capitalized on our experiences as we explored the efficiency of these strategies in teaching the four medical school courses Behavioral Science, Psychopathology, Promotion and Maintenance of Health, and Cardiovascular in the Spring and Fall semesters of 2020. These four courses were interrupted or severely affected by the COVID-19 pandemic and had to be shifted toward online-only teaching and have been evaluated by students. Following the recommendations and feedback of our students, we discuss several strategies to alleviate the extraneous load for our students.

## Alleviating Extraneous Load

As highlighted above, extraneous load is generated by the means of instruction and is not essential to comprehending the information (Sweller, 2010). However, it occupies cognitive resources and can overwhelm the WM (Stillwell et al., 2017; Jordan et al., 2020). Building upon our recent work (Mohiyeddini, sub.), we decided to purposefully alleviate the extraneous load for our students when shifting toward online-only teaching. We utilized and adapted the following five well-researched principles of CLT (van Merriënboer and Sweller, 2010).

1. *Split attention principle: this principle suggests providing one integrated source of information, avoiding temporal split attention (sources distributed in time), and/or spatial split attention (sources distributed in space).*

We anticipated that two different clusters of information produced by the COVID-19 learning environment might overwhelm each individual’s attention, challenge their internal locus of control (Abdalla et al., 2019), and create confusion: *Firstly*, we provided information related to how to create a functioning and efficient learning environment at home. To manage and integrate this information and enhance the internal locus of control (Sigurvinsdottir et al., 2020), we instituted a department-wide shared team communication platform (Slack*^R^*)^1^ at the beginning of COVID-19 pandemic to communicate issues related to the pandemic following empirical evidence that show access to valid and reliable information reduces ambiguity and anxiety (Stamenkovic et al., 2018; Sancak and Akal, 2019). We used our departmental Slack workspace in order to integrate information regarding students’ briefing, teaching, supervision, and mentoring into one tool. *Secondly*, we sought to manage COVID-19 related health- and safety information. Therefore, we thoughtfully engaged in current information and provided it with high-fidelity. To deal with this challenge, we developed a “COVID-19 updates” Slack channel to share information updates both from within our own health system as well as national/scientific news. This channel offered the most up-to-date scientific news, free from speculations and online rumors. By extension, this channel was used to reduce fear and confusion amongst relatives, friends, and communities of our students by providing a trusted information source.

2. *Worked example principle: this strategy suggests providing learners with worked examples that offer a full solution that learners may cautiously study and comprehend.*

During our orientation to shift toward online-only education, we developed several “how to” documents and offered online support at the beginning of the transition period. Further, we offered students several workshops for online education. These courses were delivered by several experts in online teaching who were able to reduce many of the “unknowns” for the students by providing detailed worked examples of how to be a successful participant in online learning.

As an effort to create an effective online learning environment for virtual team-based learning (TBL) activities, by using a shared online assignment platform we ensured that appropriate instruction and support were provided to help teams complete the assignments both effectively and efficiently. For instance, students were provided with guides for how to use the video conferencing tools and the learning management system for quizzes and application exercises. In addition, they were required to attend live synchronous ungraded TBL sessions to practice in a safe and risk-free environment. To decrease further extraneous cognitive load, we followed literature-based guidelines for TBL implementation. For example, we used a highly recommended software tool specifically designed for synchronous online TBL (InteDashboard*^R^* Inc., Singapore).^2^ This online tool automates all the TBL components (pre-class preparation, readiness assurance, and application exercises) and TBL peer feedback as well. Students conveniently access InteDashboard from within our Learning Management System (Moodle and Zoom).

3. *Completion principle: “This principle suggests substituting conventional tasks with completion tasks which provide a partial solution that must be completed by the learners”* (van Merriënboer and Sweller, 2010, p. 90*).*

TBL sessions were delivered during the curriculum using InteDashboard. This facilitated delivery of partial solutions during some TBL sessions. For poor-performing students who required remediation of a course, synchronous didactic lectures were offered in order to work closely with those students and concentrate on deficiencies in learning rather than on those areas in which the student had already demonstrated mastery. Interactive approaches were encouraged, such as flipped classroom and dialogical narrative approaches.

4. *Modality principle: this principle suggests providing a narrated text to a visual source (multimodality) instead of combining the visual source with an explanatory text in writing (unimodal).*

We solicited narrated guidelines from students to use digital guides for online classes. Furthermore, our faculty created narrated demonstrations of available tools illustrating best practices in online teaching. In addition, laboratory sessions (e.g., in pathology) in the Cardiovascular course were presented using narrated slides supported by brief video clips.

5. *Redundancy principle: Replace multiple self-contained sources with one source of information.*

We investigated all available technology options already in place within our curriculum (including Moodle, WebEx, USeeYou, Google Hangouts, and Panopto lecture recording). While we created one page ‘‘How-to’’ guides for less frequently used software tools, we have excelled in the curation of stand-alone and vetted resources for students use. WebEx was used for synchronous instruction with the entire class, with a feature to allow break-out sessions to facilitate small group work (which could be moderated by instructors). Reassembly of these small groups was also useful for collective debriefings. YouSeeU (YouSeeU*^R^* Inc., Loveland Co.)^3^ was already familiar to the students as they had been using it routinely for reflections in courses prior to COVID-19. Google Hangouts (Google*^R^* Inc., Mountain View, CA, United States)^4^ was introduced to students as a platform to mediate small group discussions and group work using video conferencing and screen-sharing. Lastly, Panopto (Panopto Inc., Pittsburgh, PA, United States)^5^ was also familiar to students as it was the software that was used for live lecture capture before COVID-19 and is now used for recording of asynchronous sessions by faculty for students. For instance, in the Promotion and Maintenance of Health course, a service-learning program was introduced to students in the first 2 weeks. Prior to the COVID-19 pandemic, sources of information were accessible to students as written instructions or as part of in-person instruction of site directors. This year, students were provided short video presentations of each site director to introduce community partner organizations. This consolidated presentation replaced what was previously obtained from multiple sources. Following student team commitments to serve with individual community partners, online training was made available online. In this way students were engaged in team projects aligned to the goal-free principle.

## Discussion and Conclusion

Most recently, Wilcha (2020) presented a systematic review of 39 articles that provide insight how several medical schools successfully implemented a variety of web-based resources and developed novel interactive forms of virtual learning for their students. However, our analysis of these articles and our extensive database searches in PubMed, Eric, PsycINFO, and PsycARTICLES revealed that our paper seems to be the first that discusses the applicability of Cognitive Load Theory in shifting toward online learning. This paper adds to our knowledge on the CLT, online education, and virtual collaborations among students for several reasons:

First, this paper shows that psychological theories can be applied to provide guidance in managing a non-voluntary process of change that was forced upon an academic institution and its students. The rationale provided in this paper could be used to develop, maintain, and enhance online teaching, supervision, and research activities during normal times as well. The digital nature of our socially distant world requires training and experience with online learning. This approach could also be used to proactively develop contingency plans and toolboxes to maintain and facilitate learning in dealing with national and international disasters in the future.

Second, our theory-driven approach provides a novel avenue to enhance the quality of online learning along with implementing purely technology-based teaching aids such as narrated PowerPoint presentations (Hampton et al., 2017), recorded lectures (Broussard and Wilson, 2018), online web-based tools (Wilson et al., 2021), use of videos (Forbes et al., 2016), using blogs (Kaup et al., 2020), and flipped classrooms (Flugelman et al., 2021).

Third, our adaptation of the CLT principles to reduce the extraneous load (such as split attention principle) rests on the assumption that these principles may serve to enhance the psychological environment for learners by reducing fear and worrisome-related thoughts because of exposure to scientifically questionable information and suggestions. In addition, our approach highlights that online education can capitalize on and profit from educational and psychological theories on learning to provide theory-based guidelines to students. Fourth, we believe our abrupt, but organized move toward online-only teaching may have enhanced the perception of the internal locus of control among students (Abdalla et al., 2019; Boyraz et al., 2019) by enhancing the sense of personal control over the situation. Furthermore, we assume that holding an early in-person workshop to demonstrate usage of online platforms might help our students to prevent a sense of helplessness, by demonstrating that our department was both theoretically and technically prepared to provide support through the COVID-19 pandemic (Lester, 2012; Shaw, 2020). Sixth, our approach can foster the quality of learning during normal times. Cultivating a culture of online collaborations can be beneficial to maintaining and fostering national and international collaborations among students as well. Last, our approach delivers a theory-based example of how to create a virtual collaborative space for educational activities among students which may add to their traditional face-to-face scholarly skills and resources.

As is inherent to the nature of an opinion article, this paper suffers from two major limitations: (1) Our paper does not provide empirical evidence to support our claims and suggestions. Instead, we relied on empirical evidence that have supported the principles of CLT during normal times and apply those to the times of the COVID-19 pandemic. (2) This paper reflects theory-driven changes that were implemented on an individual academic institution’s actions to enhance students’ learning and, hence, contains bias and lacks external validity. (3) By applying an empirically investigated theory such as CLT, we have also adopted the shortcomings of this theory. For instance, it is still unclear how cognitive load can be measured (Holton, 2009). CLT neglects interindividual differences by learning and assumes that instructional materials can be the same for all learners. In addition, Moreno (2010) criticizes a lack of conceptual clarity regarding terms such as cognitive load, mental load, and mental effort. In addition, Reif (2010) highlighted that minimizing cognitive load doesn’t constitute necessarily a more efficient learning condition as a low cognitive load may result in boredom, ultimately diminishing a learner’s motivation to learn. Another key shortcoming of CLT is that it might be immune to empirical falsification. For instance, it seems challenging to experimentally vary the level of cognitive load independent from learning performance of individuals as CLT equates poor learning performance with high levels of cognitive load. Relatedly, de Jong (2010) emphasizes that germane cognitive load is a *post hoc* explanation that lacks theoretical basis. However, remote teaching seems to offer itself as an inherent component of future higher education. Therefore, we aim to provide empirical evidence to highlight the importance of theory-driven online learning strategies to minimize the cognitive load for students.

## Author Contributions

CM provided the idea and theoretical background and designed the manuscript. TT, SK-E, JG, IH, and SL contributed to the different parts of the manuscript. All authors contributed to the article and approved the submitted version.

## Conflict of Interest

The authors declare that the research was conducted in the absence of any commercial or financial relationships that could be construed as a potential conflict of interest.

## Publisher’s Note

All claims expressed in this article are solely those of the authors and do not necessarily represent those of their affiliated organizations, or those of the publisher, the editors and the reviewers. Any product that may be evaluated in this article, or claim that may be made by its manufacturer, is not guaranteed or endorsed by the publisher.

## Footnotes

1
www.slack.com

2
www.intedashboard.com

3
www.youseeu.com

4
https://meet.google.com/

5
www.panopto.com
